# Predictors of Neoplasia in Colonic Wall Thickening Detected via Computerized Tomography

**DOI:** 10.7759/cureus.10553

**Published:** 2020-09-20

**Authors:** Cengiz Karacin, Sema Türker, Tulay Eren, Goksen Inanc Imamoglu, Kemalettin Yılmaz, Yusuf Coskun, Serra Ozbal Gunes, Fevzi Sökmen, Dogan Yazilitas, Zahide Şimşek, Mustafa Altınbaş

**Affiliations:** 1 Medical Oncology, Dr. Abdurrahman Yurtaslan Oncology Training and Research Hospital, Ankara, TUR; 2 Medical Oncology, Diskapi Yildirim Beyazit Training and Research Hospital, Ankara, TUR; 3 Gastroenterology, Diskapi Yildirim Beyazit Training and Research Hospital, Ankara, TUR; 4 Radiology, University of Health Sciences, Diskapi Yildirim Beyazit Training and Research Hospital, Ankara, TUR; 5 Internal Medicine, Dr. Abdurrahman Yurtaslan Oncology Training and Research Hospital, Ankara, TUR; 6 Medical Oncology, Diskapi Yildirim Beyazit Research and Education Hospital, Ankara, TUR

**Keywords:** colonic wall thickening, computerized tomography, neoplasia, colonoscopy

## Abstract

Introduction

Colonic wall thickening (CWT) is frequently observed incidentally via abdominal computerized tomography (aCT). Although the general approach to evaluating incidental CWT is a colonoscopic examination, there is a lack of definitive recommendation guidelines. Thus, we aimed to determine neoplasia rates and identify the factors predictive of neoplasia via colonoscopic examinations of patients with CWT incidentally diagnosed via aCT.

Methods

We retrospectively reviewed 5,300 colonoscopy reports. A total of 122 patients who had CWT incidentally observed via aCT were included in the study. CWT was graded as mild (3-5 mm), moderate (6-12 mm), or severe (≥12 mm). A logistic regression model was used to determine the predictive factors for neoplasia.

Results

The mean age of the patients was 60 years, and abnormal findings were noted in 52% of the colonoscopies. Neoplastic lesions were detected in 24 patients (19.6%), while colon adenocarcinoma was detected in 8 patients (6.5%). Multivariate analysis showed that moderate-severe, focal, and asymmetric CWT were independent factors for predicting neoplasia (p=0.049, p=0.033, and p=0.018, respectively).

Conclusion

Pathological findings can be noted via colonoscopic examination in cases of incidental CWT; therefore, patients with moderate-severe, focal, or asymmetric CWT require colonoscopic examination for the purpose of detecting neoplasia.

## Introduction

Abdominal computerized tomography (aCT) is widely used for the diagnosis of intra-abdominal pathologies [1]. Colonic wall thickening (CWT) is a common incidental aCT finding [1,2]. Although clinicians’ general approach to CWT is to perform colonoscopic examination to determine the underlying pathology, there is a lack of definitive recommendation guidelines [2]. Patients with CWT have underlying infection, inflammation, ischemia, or neoplasia [3]. CWT can be detected in diseases such as cirrhosis, heart failure, and hypoalbuminemia due to intestinal wall edema [4]. Premalignant-malignant lesion rate is reported to be 15%-65% in CWT studies [2]. A few studies have shown that neoplastic lesions cause significant and focal thickening, based on aCT, but no definitive markers predicting neoplasia have been identified.

Early diagnosis of colorectal cancer improves overall survival [5], and excision of precancerous polyps prevents colorectal cancer formation [6]. Therefore, colonoscopic examination of CWT is important for the detection of neoplastic lesions. Yet, both patients and clinicians have difficulty deciding whether or not to perform/undergo colonoscopy because of its invasive nature and the associated risk of complications.

Most studies on colonoscopy results in CWT patients include small study populations [2,3,7]. In addition, the degree, characterization, and localization of wall thickening are not reported [2,7,8]. Therefore, the present study aimed to determine the neoplasia rate and identify the factors predictive of neoplasia via colonoscopic examination in a homogeneous group of patients with CWT incidentally diagnosed via aCT.

## Materials and methods

Patients

Colonoscopy reports of 5,300 patients who underwent colonoscopy between 01/01/2013 and 01/01/2017 were retrospectively reviewed. A total of 122 patients with CWT incidentally diagnosed via aCT, who met the study inclusion criteria, were included in this study. Inclusion criteria were age ≥18 years, CWT ≥3 mm incidentally found via aCT, and those undergoing colonoscopy within four weeks of aCT. Patients with clinical conditions that can make CWT, such as cancer, anemia, cirrhosis, hypoalbuminemia, weight loss, heart failure, inflammatory bowel disease (IBD), inadequate colonoscopic examination, and inadequate filling of the intestinal lumen with the CT contrast material, were excluded.

Study design

All CT images were retrospectively reviewed by an experienced radiologist, and CWT was graded as mild (3-5 mm), moderate (6-12 mm), and severe (≥12 mm) [9]. CWT localization was categorized as right and left. The left colon included the distal of the transverse colon, splenic flexure, descending colon, sigmoid colon, and rectum. The right colon included the proximal of the transverse colon, ascending colon, and cecum.

Each patient’s age, gender, medical history, and pathology report were obtained from an archive and electronic data system. The colonoscopy and pathology reports were classified as normal or abnormal. Abnormal pathology reports were categorized as neoplastic or non-neoplastic.

The study was approved by the Ethics Committee of Dışkapı Yıldırım Beyazıt Training and Research Hospital (No: 39/19, 06/12/2017). All the methods in the present study were carried out in accordance with guidelines of the Declaration of Helsinki. All participants provided written informed consent.

Statistical analysis

Data obtained in the study were analyzed statistically using IBM SPSS Version 20 software (Armonk, NY: IBM Corp). Each parametric variable was given as a mean with standard deviation, whereas the categorical variables were given as proportions. A Student t-test was used to compare the parametric variables, whereas a chi-square or Fisher’s exact test was used to compare the categorical groups. A binary logistic regression model was used to find the independent predictive factors for the neoplasia. A p-value <0.05 was considered statistically significant.

## Results

A total of 122 patients were included in this study, and their mean age was 60±13.8 years. The majority of the patients were male (54.1%). In terms of CWT localization, 73.8% of the study participants had CWT on the left colon. The other characteristic features of their CWT are given in Table 1.

**Table 1:** Demographic features of the patients and characteristic features of the CWT CWT: colonic wall thickening

Variables		Values
Age, mean±SD		60±13.8
Gender, male, n (%)		66 (54.1)
CWT localization		
	Right colon, n (%)	32 (26.2)
	Left colon, n (%)	90 (73.8)
Degree of CWT		
	Mild, n (%)	93 (76.2)
	Moderate, n (%)	19 (15.6)
	Severe, n (%)	10 (8.2)
Symmetry of CWT		
	Symmetric, n (%)	94 (77.0)
	Asymmetric, n (%)	28 (23.0)
Length of CWT		
	Focal, n (%)	54 (44.3)
	Segmental, n (%)	54 (44.3)
	Diffuse, n (%)	14 (11.4)

Colonoscopy findings were normal in 59 (48.3%) patients. Polypoid lesions were noted in 23 (18.9%) of the 122 patients, ulcerations in 21 (17.2%), ulceronodular lesions in 15 (12.3%), and tumor masses in 4 (3.3%). Non-specific colitis was the most frequent histopathological evaluation result of abnormal colonoscopic findings. Among the 63 patients with abnormal colonoscopy findings, 16 (25.4%) had adenomatous polyps and 8 (12.9%) had colon cancer. Histopathological findings in the patients with abnormal colonoscopy findings are shown in Table 2.

**Table 2:** Histopathological findings of the patients with abnormal colonoscopy

	n=63 (%)
Non-specific colitis	27 (42.9)
Inflammatory bowel disease	7 (11.1)
Hyperplastic polyp	5 (7.9)
Adenomatous polyp	16 (25.4)
Colon adenocarcinoma	8 (12.7)

**Figure 1:**
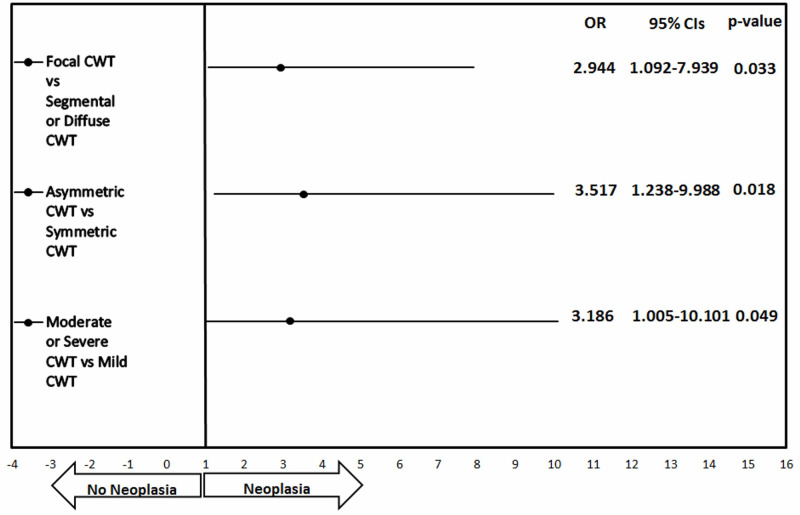
The forest plot shows the OR for neoplasia and 95% CI (I bars) for patients with CWT detected on CT, according to degree, length. and symmetry of CWT. CWT: colonic wall thickening

Neoplastic lesions were detected in 24 (19.6%) of the 122 patients, whereas colon adenocarcinoma was detected in 8 patients (6.5%). Comparison of demographics and CWT findings of the patients with and without neoplasia is shown in Table 3. There was no significant difference between the groups in terms of age and gender. According to univariate analysis, the rates of moderate-severe, focal, and asymmetric CWT were higher in those with neoplasia. Multivariate analysis showed that moderate-severe, focal, and asymmetric CWT were independent factors predictive of neoplasia (p=0.049, p=0.033, and 0.018, respectively, Figure 1).

**Table 3:** The comparison of demographic features and CWT findings of neoplasia and non-neoplasia groups CWT: colonic wall thickening

	Neoplasia n (%)	Non-neoplasia n (%)	P value
Age ≥50 years, n (%)	14 (58.3)	48 (49.0)	0.411
Gender, male, n (%)	13 (54.1)	53 (54.0)	0.985
CWT localization, left colon, n (%)	18 (75.0)	72 (73.5)	0.879
Moderate-severe CWT, n (%)	8 (33.3)	14 (14.3)	0.040
Asymmetric CWT, n (%)	10 (41.7)	18 (18.4)	0.015
Focal CWT, n (%)	15 (62.5)	36 (36.7)	0.022

## Discussion

The present study investigated the factors predictive of neoplastic lesions in patients with CWT incidentally detected via aCT. The present findings show that focal, moderate-severe, and asymmetric wall thickening are predictive of neoplastic lesions.

The reported rate of colorectal cancer in patients with CWT is 14%-27%, and most of the cancer patients included in such studies were asymptomatic. Moraitis et al. observed a neoplasia rate of 23% and a colon cancer rate of 14% in their small study [3]. They also noted that 80% of patients with colon cancer did not have gastrointestinal symptoms. Tellez-Avila et al. reported a colon cancer rate of 20%, and showed that colon cancer was higher in anemic patients [10]. Patel et al. noted neoplasia in 13% and colon cancer in 8% of the patients with CWT [11]. Uzzaman et al. reported a neoplasia rate of 35.7% and a cancer rate of 21.8% in patients with CWT [1]. They also reported that the neoplastic lesion rate was higher in patients with rectal bleeding (30.5%). A prospective study by Khairnar et al. observed a cancer rate of 11.7% in CWT patients and showed that irregular or moderate-severe wall thickening can predict cancer [12]. In our study, we detected the neoplasia rate of 19.6% and the cancer rate of 6.5%. Unlike other studies, we excluded the patients with malignant symptoms such as rectal bleeding and weight loss.

Except in rare cases, long segmental wall thickening is associated with benign conditions [3]. Focal wall thickening usually indicates a malignancy or an inflammatory process [13]. CT findings of gastrointestinal tract tumors are usually focal wall thickening [3]. Tapasvi et al. showed that focal wall thickening detected in CT is associated with malignancy. They also reported a malignancy rate of 84% in patients with focal wall thickening versus 54% in those without focal wall thickening [14]. In our study, malignancy rate was higher in those with focal wall thickening (29.4% vs 12.7%). We also showed that focal wall thickening is an independent predictive factor for neoplasia as a result of multivariate analysis.

Bharucha et al. studied CWT in three groups, according to their grade [9]. Wall thickness was defined as mild (3-6 mm), medium (6-12 mm), and severe (>12 mm). Recent studies showed that mild wall thickening is generally associated with benign conditions, whereas severe wall thickening is associated with malignant conditions. However, the majority of these studies included patients with small bowel wall thickening. Khairnar et al. showed that irregular and moderate-severe wall thickening can predict cancer [12]. Similarly, in our study, neoplasia was more common in those with moderate-severe CWT. Moreover, moderate-severe CWT was observed to be an independent factor predictive of neoplasia.

Symmetrical wall thickening is observed in cases of inflammation, infection, edema, ischemia, and submucosal bleeding [3]. Symmetrical wall thickening, except for lymphoma, is associated with benign conditions. Although asymmetric wall thickening is usually a marker of malignancy, it can sometimes be seen in patients with such non-malignant conditions, such as intestinal tuberculosis and IBD [4,15]. In our study, the asymmetric wall thickening rate was 41% in the patients with neoplasia versus 18% in those without neoplasia. In addition, asymmetric wall thickening was an independent factor predictive of neoplasia.

Earlier studies show that the correlation between CWT and colonoscopic findings is associated with CWT localization. Cai et al. observed abnormal colonoscopic findings in 81% of patients with rectosigmoid wall thickening and 13% of those with cecal wall thickening [16]. Khairnar et al. reported that abnormal colonoscopy findings were more common in patients with CWT localized in the left colon, as opposed to the right colon [12]. Uzzaman et al. noted that wall thickening detected in the transverse colon was associated with cancer [1]. In our study, similar to other studies, we found more abnormal findings in the colonoscopic examination of the left CWT. However, there was no significant difference between the right and left colon in terms of the presence of neoplasia.

Akbas et al. retrospectively analyzed the colonoscopic evaluations of CWTs reported in aCT for any reason in their studies [17]. They have focused on hemoglobin, neutrophil-lymphocyte ratio (NLR), and mean platelet volume (MPV) of patients with CWT and contribute to the separation of benign and malignant pathologies. In our study, we did not examine NLR and MPV, but we defined anemia, a sign of malignancy, as an exclusion criterion in terms of not affecting the results.

This study has some limitations, including a retrospective design. Due to the lack of colonoscopy in all patients with CWT incidentally detected via aCT, the present findings cannot be generalized to all patients with incidental CWT. In addition CTs were re-evaluated by only one radiologist, which might have had a negative effect on objective interpretation of CWT characteristics.

## Conclusions

Neoplasms constitute a significant percentage of pathologies that can cause CWT. Patients with moderate-severe, focal, and asymmetric CWT should be evaluated colonoscopically. The results obtained in this study can be supported by studies in which a prospective colonoscopic evaluation of CWT will be performed. 
